# Brief research report: autistic traits modulate the rapid detection of punishment-associated neutral faces

**DOI:** 10.3389/fpsyg.2023.1284739

**Published:** 2023-11-21

**Authors:** Akie Saito, Wataru Sato, Sakiko Yoshikawa

**Affiliations:** ^1^Psychological Process Research Team, Guardian Robot Project, RIKEN, Kyoto, Japan; ^2^Field Science Education and Research Center, Kyoto University, Kyoto, Japan; ^3^Faculty of Art and Design, Kyoto University of the Arts, Uryuuzan, Kyoto, Japan

**Keywords:** autistic traits, autism, visual search, neutral faces, emotional value, learning

## Abstract

Speedy detection of faces with emotional value plays a fundamental role in social interactions. A few previous studies using a visual search paradigm have reported that individuals with high autistic traits (ATs), who are characterized by deficits in social interactions, demonstrated decreased detection performance for emotional facial expressions. However, whether ATs modulate the rapid detection of faces with emotional value remains inconclusive because emotional facial expressions involve salient visual features (i.e., a U-shaped mouth in a happy expression) that can facilitate visual attention. In order to disentangle the effects of visual factors from the rapid detection of emotional faces, we examined the rapid detection of neutral faces associated with emotional value among young adults with varying degrees of ATs in a visual search task. In the experiment, participants performed a learning task wherein neutral faces were paired with monetary reward, monetary punishment, or no monetary outcome, such that the neutral faces acquired positive, negative, or no emotional value, respectively. During the subsequent visual search task, previously learned neutral faces were presented as discrepant faces among newly presented neutral distractor faces, and the participants were asked to detect the discrepant faces. The results demonstrated a significant negative association between the degrees of ATs and an advantage in detecting punishment-associated neutral faces. This indicates the decreased detection of faces with negative value in individuals with higher ATs, which may contribute to their difficulty in making prompt responses in social situations.

## 1 Introduction

Rapid detection of emotional faces underlies the emotion recognition processes, which are critical for understanding others’ emotions to establish interpersonal relationships. Prior studies have reported that emotional facial expressions are rapidly detected by typically developing individuals in visual search tasks (Hansen and Hansen, 1988; Eastwood et al., 2003; Williams et al., 2005). Similarly, neutral faces that had acquired emotional value via associative learning have been demonstrated to be rapidly detected by typically developing individuals (Saito et al., 2022). Acquired emotional value is assumed to attract attention, thereby facilitating the detection of value-associated neutral faces, as in the case of emotional facial expressions (Bliss-Moreau et al., 2008; Gupta et al., 2016).

Individuals with high autistic traits (ATs) are reportedly impaired in the rapid detection of emotional facial expressions in a visual search task, whereby individuals categorized as having high ATs took longer to detect happy facial expressions than those with low ATs (Sato et al., 2017). ATs are a broader phenotype of autism spectrum disorder (ASD) (Baron-Cohen et al., 2001), associated with deficits in social–emotional interactions (American Psychiatric Association, 2013). ATs exist on a continuum among the general population, and their severity can be measured using the Autism Spectrum Quotient (AQ) (Baron-Cohen et al., 2001). Consistent with the findings of the previous visual search research, several studies have demonstrated an impaired attentional shift to emotional expressions in individuals with high ATs (Poljac et al., 2013; Lassalle and Itier, 2015) and ASD (Uono et al., 2009; Farran et al., 2011; Van der Donck et al., 2020; Macari et al., 2021).

However, the extent to which ATs modulate the detection speed for faces with emotional value in a visual search task remains unclear. Aside from their emotional value, salient visual features in emotional expressions (e.g., a U-shaped mouth in a happy expression) promote visual attention to the expressions (Calvo and Nummenmaa, 2008), suggesting that salient visual features as well as emotional value might facilitate the rapid detection of emotional facial expressions. Although the detection advantage for emotional facial expressions in photographed faces has been found compared to the controlled photographed faces that have comparable featural changes to those of real emotional facial expressions among neurotypical young adults (Sato and Yoshikawa, 2010), such control of visual features may not completely exclude the effects of visual factors on face processing because these control faces differ from faces showing emotional facial expressions in terms of processing holistic information in the faces (Tanaka and Farah, 1993; Sato and Yoshikawa, 2010). In contrast, neutral faces that acquired emotional value via learning have no salient visual features (i.e., excluding low-level visual confounds) and allow the processing of holistic information of the faces similar to that of emotional facial expressions, since neutral faces are frequently encountered in our daily life as faces displaying neutral emotion. It is therefore possible to say that neutral faces that have acquired emotional value via associative learning may be the best controlled faces to investigate the rapid detection of facial expressions with emotional meaning. Furthermore, autistic individuals are more likely to rely on visual features in detecting emotional facial expressions in a visual search task than neurotypical individuals (Ashwin et al., 2006; Isomura et al., 2014). Given that individuals with higher ATs show similar qualitative autistic symptomatology, the contribution of visual features to the rapid detection of emotional facial expressions is greater in individuals with higher ATs than in those with lower ATs, which further strengthens the need to examine the modulation of ATs on visual search performance with the use of faces that contain emotional value but are free from confounding visual features. Thus, the use of neutral faces associated with learned emotional value rather than emotional expressions themselves (Saito et al., 2022) may help clarify the modulating effect of ATs on the detection speed for faces with emotional value. No studies have yet examined the rapid detection of neutral faces associated with emotional value in visual searches in terms of ATs. The only relevant study, which used an eye-tracking methodology, has reported decreased value-driven visual attention to social stimuli (faces) among children with ASD (Wang et al., 2020).

In this study, we examined whether ATs modulate the detection of neutral faces associated with emotional value via learning. Specifically, we investigated the potential relationship between ATs and the speed with which participants detected neutral faces associated with emotional value. Based on prior studies suggesting diminished allocation of visual attention to value-associated and emotional faces among individuals with ASD and high ATs (Poljac et al., 2013; Lassalle and Itier, 2015; Sato et al., 2017; Van der Donck et al., 2020; Wang et al., 2020; Macari et al., 2021), we hypothesized a negative association between AT severity and the ability to rapidly detect neutral faces with emotional value.

## 2 Methods

### 2.1 Participants

The data from the final sample of our previous experiments (Saito et al., 2022, submitted) were analyzed (35 females and 37 males, mean ± *SD* age = 22.1 ± 1.7 years). The participants were Japanese undergraduate or graduate students with normal or corrected-to-normal vision. The sample size was determined based on an *a priori* power analysis using G*Power software 3.1.9.2 (Faul et al., 2007). Assuming six measurements and three independent variables for the linear multiple regression, with an α level of 0.05 (one-tailed), power of 0.80, and effect size *f* ^2^ of 0.15 (medium), 43 participants were found to be required. The participants were compensated for their participation and provided written informed consent.

### 2.2 Materials

#### 2.2.1 Apparatus

Stimuli were displayed on a 19-inch monitor (HM903D-A; Iiyama, Tokyo, Japan), with a refresh rate of 150 Hz and a resolution of 1,024 × 768 pixels, controlled by Presentation 14.9 software (Neurobehavioral Systems, San Francisco, CA, USA) on a Windows computer (HP Z200 SFF; Hewlett-Packard, Tokyo, Japan). Responses were collected via a response box (RB-530; Cedrus, San Pedro, CA, USA) with a 2–3-ms reaction time (RT) resolution.

#### 2.2.2 Stimuli

**Figure 1:**
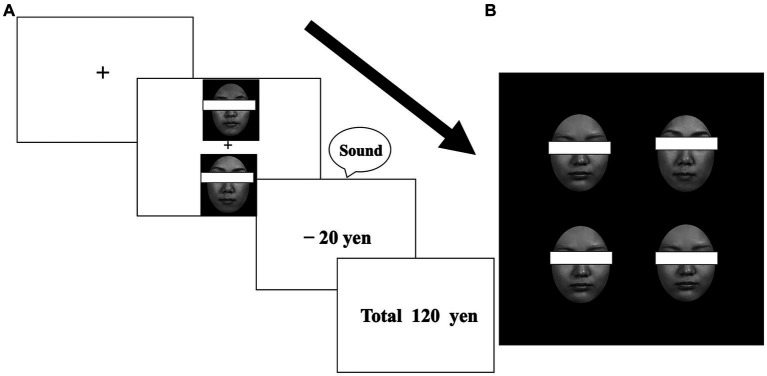
Example stimuli from the learning **(A)** and visual search **(B)** tasks. Participants were required to select one face to maximize their earnings **(A)**. After learning, participants were required to detect one different face among distractor faces (for target-present trials) **(B)**. Faces were not covered with eye masks in the experiment.

Six grayscale photographs of neutral faces (targets) and one distractor neutral face were selected for each sex (14 faces in total) from a database of Japanese individuals (Sato et al., 2019; Figure 1B). To select nonspecific target faces, a preliminary rating experiment was conducted with 14 participants (7 females; mean ± *SD* age = 23.7 ± 2.3 years), none of whom participated in the main experiment. Participants were presented with 65 neutral faces selected from the database and rated the faces on a 5-point scale of distinctiveness and attractiveness. As a result, 14 faces that were rated as relatively neutral in terms of distinctiveness and attractiveness (within the range of the mean ± 1 *SD*) were selected. All stimuli were adjusted for light and shade using Photoshop 5.0 (Adobe, San Jose, CA) and controlled for attractiveness and distinctiveness. The mean luminance of the stimuli was equalized using MATLAB R2017b (MathWorks, Natick, MA). Each face stimulus was cropped to an ellipsoid frame to exclude distinctive factors (e.g., hairstyle, facial contours), with subtended visual angles of 3.5° horizontally and 4.5° vertically.

##### 2.2.2.1 Associative learning task

Three pairs of neutral faces of each sex were used. For each sex, one pair was allocated to one of the three value-type conditions (reward, punishment, or zero outcomes). In the reward and punishment conditions, one face from each pair was designated as the target; choosing the target face yielded a monetary reward (20 yen increase for each trial) in the reward condition or incurred a monetary loss (20 yen decrease for each trial) in the punishment condition, with high validity (80% probability, otherwise, 20% zero outcomes). The contingency was reversed for the non-target faces in each pair; choosing a non-target face yielded monetary reward or loss with low validity (20% probability, otherwise, 80% zero outcomes). In the zero-outcome condition, one face was assigned to a target, but monetary outcomes were always zero (choosing a non-target face also yielded no outcomes) (Figure 1A). Approximately half (*n* = 37) of the participants, who consisted of female and male participants, were shown neutral male faces, and the remaining participants (*n* = 35), who consisted of female and male participants, were shown neutral female faces. Across the participants, the allocation of target faces in each condition was counterbalanced. Each participant performed the learning task, experiencing any of the possible 24 patterns of combinations. The face pairing was fixed throughout the task so that each face always appeared with its partner.

##### 2.2.2.2 Visual search task

Only target faces that each participant had seen during learning were used, along with one neutral distractor face. Four faces appeared simultaneously in a square configuration (11.0° × 11.0°) on the display, with each face appearing in one of four positions at 4° intervals. One target and three identical neutral distractor faces appeared for target-present trials, and four identical distractor faces were displayed for target-absent trials. In the main experiment, each target face appeared an equal number of times in all four positions (i.e., a total of 32 appearances per target across the four blocks). To control detection speed among target faces, a preliminary experiment examined whether target faces were detected equally when they were presented as discrepant faces for target-present trials in visual searches, by implementing the same experimental procedure as in the main experiment of the visual search task. The analyses of the preliminary experiment revealed that the detection speed did not differ significantly among target faces in the visual search task [*F*(5,35) = 2.18, *p* = 0.079 for male faces; *F*(5,30) = 1.05, *p* = 0.41 for female faces].

#### 2.2.3 Procedure

The participants were seated 80 cm from the screen in a chair with a fixed chin rest in a dimly lit, soundproofed room (Science Cabin, Takahashi Kensetsu, Tokyo, Japan). The participants completed an associative learning task before the visual search task as part of a larger study involving other cognitive tasks and questionnaires.

##### 2.2.3.1 Associative learning task

The participants were instructed to choose a face from each pair based on their “gut feeling” by pressing the corresponding button. The goal was to maximize their earnings. The participants were told that any money they earned would be paid at the end of the experiment and were encouraged to do their best to earn money. Each pair of faces appeared simultaneously in the two positions at the center of the screen in a pseudorandom order. After a 0.9° × 0.9° fixation cross had been presented for 500 ms, one face appeared 2.5° above the cross and the other face appeared 2.5° below the cross. Following the selection, a “price” message appeared (+20 yen, −20 yen, or 0 yen), with a sound indicating whether the answer was correct (with no sound for the “0 yen” message). The running total of yen earned was displayed for 1,800 ms. Each pair of faces appeared on the screen 10 times per block (i.e., a total of 30 trials). The order in which each pair was presented was pseudo-randomized to prevent the consecutive presentation of the same face pairs in the same positions within each block. Thirty practice trials preceded the main experiment, which consisted of 10 blocks of 30 trials.

##### 2.2.3.2 Visual search task

Participants were informed that this task was unrelated to earning money. After the fixation cross was displayed for 500 ms, four faces were presented simultaneously. The participants were asked to indicate whether the faces were the same or different by pressing the corresponding button as quickly and accurately as possible. Response button allocations were counterbalanced across participants. Each block consisted of 24 target-present trials (eight times each for reward, loss, and zero-outcome conditions) and 24 target-absent trials (48 trials, in total). To prevent the consecutive presentation of the same targets in the same positions, the order in which trials were presented was pseudo-randomized within each block. The main experiment consisted of four blocks of 48 trials and was preceded by 24 practice trials. After the experiment, the participants were asked to report anything they noticed during the two different tasks. Then, they were debriefed.

#### 2.2.4 Questionnaires

After the visual search task, participants completed the Japanese short version of the AQ, which includes a 21-item questionnaire (Kurita et al., 2005). Because a depressive state could be a confounding factor in the investigation of AT, the Japanese version of the Beck Depression Inventory-II (BDI-II) (Nihon Bunka Kagakusha, Tokyo, Japan) was administered.

### 2.3 Data analyses

Data were analyzed by JASP 0.14.1 (JASP Team, 2020). For the learning task, selecting optimal faces in the last block (i.e., targets in the reward and non-targets in the punishment conditions) on >65% of trials of the block was considered to indicate task success, following previous studies (O’Brien and Raymond, 2012; Gupta et al., 2016). The data of the participants who succeeded in the learning task were used in the analysis of visual search speed. Preliminary analyses showed no significant correlations between the selection rates of optimal faces during learning and ATs (*r* = −0.10 and 0.02 for reward and punishment faces, respectively, *n.s*.). For the visual search task, mean RTs of the correct responses for each condition of the target-present trials were calculated after excluding responses longer than 3 s and the measurement ±2 *SD* from the mean for each participant. As an index of the detection advantage of value-associated faces, RT difference scores between the no-value and valued conditions were calculated for both the reward and punishment conditions. One participant’s RT difference scores for the punishment condition were excluded because they were outliers (> 3 *SD* from the group mean). Multiple regression analyses were then conducted with the RT difference scores (reward or punishment) as the dependent variable and ATs as the independent variable. Age, sex, and depression scores were included as covariates (effects of no interest). Based on our prediction, the effect of AT was tested using *t*-statistics (one-tailed). Partial correlation coefficients (*prs*) were reported as the effect size indices (Aloe and Thompson, 2013). Our preliminary evaluation showed that none of the covariates reached significance (two-tailed *t*-test, *p* > 0.05). Most of the participants (68/72) were categorized as minimal or mild for the Japanese version of the BDI, which was comparable with the distribution of the scores of Japanese University students (Nishiyama and Sakai, 2009). The strength of AT–RT difference scores was also compared between the reward and punishment conditions using the *F*-test for parallelism (Finney, 1964; Council of Europe, 2011). The results of these frequentist models were considered statistically significant at *p* < 0.05. In addition, we explored the above multiple regression models using Bayesian methods (Baldwin and Larson, 2017). We set the Akaike Information Criterion prior on parameters (*cf.* Caraka et al., 2021) and used the default model prior and sampling methods. We compared the above model with AT and covariates (age, sex, and depression scores) and the null model only with covariates based on the inclusion Bayes Factors and described the coefficient information for AT (*β* values and 95% credible interval [one-tailed]).

## 3 Results

**Figure 2:**
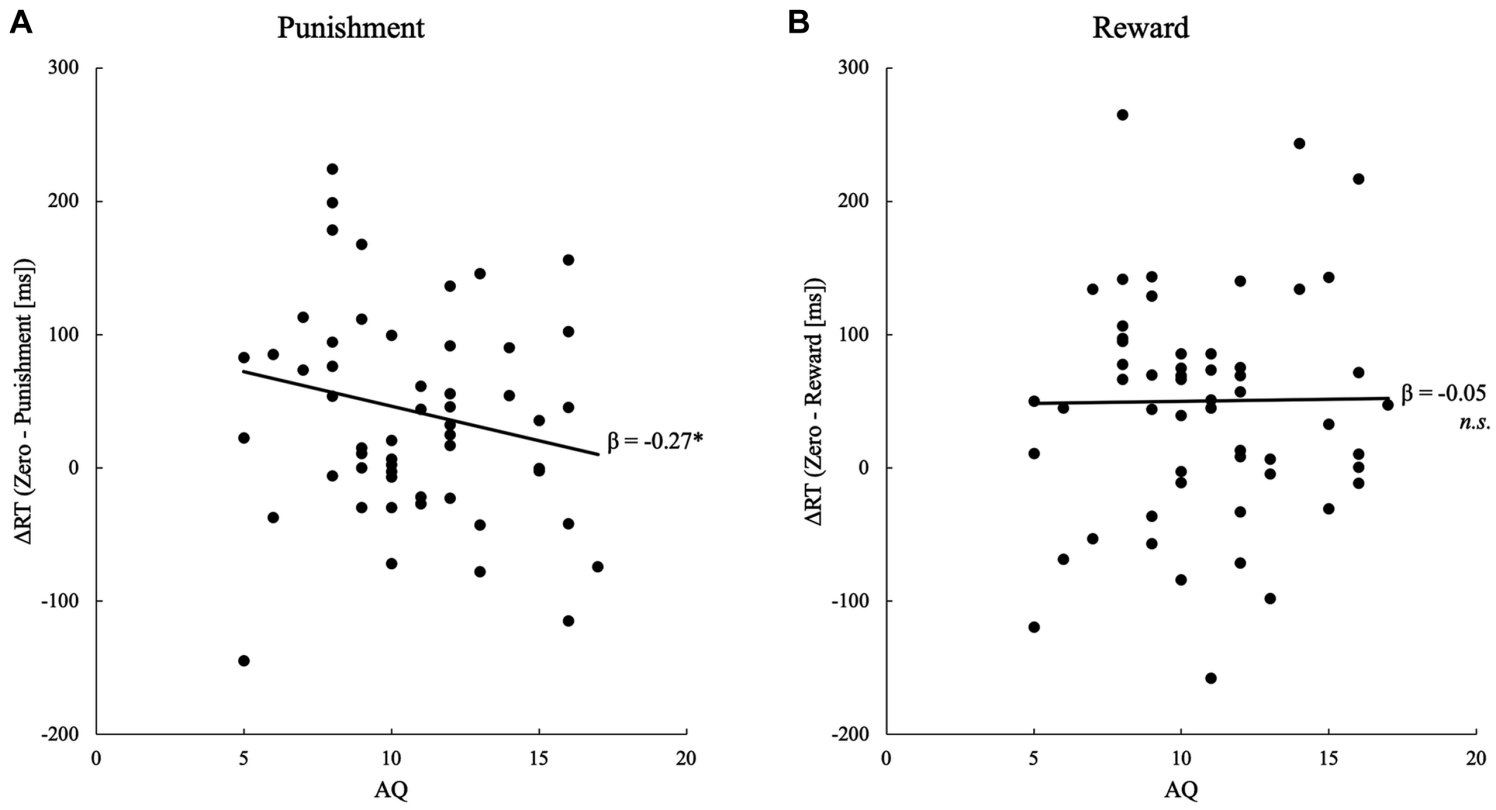
Scatterplot and regression line showing the significant relationship between the detection speed of value-associated faces and AQ scores for **(A)** punishment and **(B)** reward.

Twenty-two participants out of the 56 participants of successful learners scored above the cut-off point (12) on the Japanese short version of the AQ (Figure 2). Multiple regression analyses that tested the effect of AT on RT difference scores between the no-value and punishment conditions (i.e., “the index of advantage” of valued faces) revealed a significant negative association between the ATs and RT difference scores [*β* ± *SE* = −7.09 ± 4.13, standardized *β* = −0.26, *t* = −1.72, *p* = 0.046, *pr* = −0.24] (Figure 2). Analysis of the RT difference scores between the no-value and reward conditions showed no significant association [*β* ± *SE* = −0.22 ± 4.22, standardized *β* = −0.01, *t* = −0.05, *p* = 0.480, *pr* = −0.01] (Figure 2). The strength of the association between the AT and RT difference scores did not differ significantly between the reward and punishment conditions [*F*(1,107) = 1.22, *p* = 0.272, *η*^2^ = 0.01].

To supplement the frequentist method analysis (Baldwin and Larson, 2017), the multiple regression models noted above were analyzed using Bayesian methods. For the punishment conditions, model comparison provided anecdotal evidence for preferring the model with AT as compared to the null model (inclusion Bayes factor = 1.78). In this model, the AT coefficient was suggested to be negative and different from zero (*β* ± *SD* = −7.09 ± 4.13, 95% credible interval = [−14.02, −0.17]). For the reward conditions, model comparison showed anecdotal evidence that the model with AT was not supported as compared to the null model (inclusion Bayes factor = 0.37). In the full model, the AT was not clearly different from zero (*β* ± *SD* = −0.22 ± 4.22, 95% credible interval = [−2.77, 3.59]).

## 4 Discussion

This study examined whether ATs modulate the rapid detection of neutral faces with emotional value in a visual search task. The results showed a significant negative association between ATs and a detection advantage for neutral faces associated with punishment, indicating that the increased severity of ATs is associated with slower detection of punishment-associated neutral faces. The results suggest that acquired emotional value exerts a weaker influence on the ability to rapidly detect value-associated neutral faces among young adults with higher ATs. This result is consistent with a previous eye-tracking study reporting diminished value-driven visual attention to faces among children with ASD (Wang et al., 2020) and research demonstrating a weakened transfer of acquired social–emotional value to subsequent behavior among individuals with high autistic traits (Panasiti et al., 2016), which might be underpinned by diminished neural responses to socially rewarding stimuli found among individuals with high autistic traits (Cox et al., 2015) and those with ASD (Scott-Van Zeeland et al., 2010). Our results, along with these previous findings, could be explained by the decreased learned value placed on social stimuli by individuals with ASD as stated by the Social Motivation Hypothesis of Autism (Dawson et al., 1998, 2004; Chevallier et al., 2012).

Crucially, we were able to clarify whether ATs modulated the rapid detection of faces with emotional value as the use of neutral faces allowed us to exclude the confounding effect of visual saliency. Thus, our results indicate decreased detection performance for faces with emotional value in individuals with higher ATs, which is consistent with a previous visual search study showing impaired detection of emotional facial expressions in individuals with high ATs (Sato et al., 2017). Moreover, decreased detection of neutral faces associated with negative emotional value (punishment) among individuals with higher ATs is consistent with earlier studies demonstrating compromised attention allocation to negative emotional faces among individuals with high ATs and ASD (Uono et al., 2009; Farran et al., 2011; Poljac et al., 2013; Ghosn et al., 2019; Van der Donck et al., 2020; Macari et al., 2021). Our results particularly align with those of García-Blanco et al. (2017) and Matsuda et al. (2015), who demonstrated a relationship between the severity of social deficits and diminished visual attention allocation to negative faces in ASD. Thus, our findings extend these results to neutral faces associated with learned negative emotional value, which are not confounded by visual saliency.

Our results might suggest reduced motivation in processing negative faces pertaining to autistic traits, which is related to the Intense World Theory (Markram et al., 2007; Markram and Markram, 2010). According to this theory, individuals with ASD experience sensory stimuli as excessively intense and aversive, so they are likely to employ altered perceptions of these aversive stimuli to regulate their internal emotional experience, leading to a decline in their motivation to process negative social stimuli among individuals with high ATs and ASD (Ghosn et al., 2019; Yang et al., 2022).

Alternatively, our results might just reflect slower behavioral responses of pressing the corresponding button for neutral faces with punishment; individuals with higher ATs detected neutral faces with punishment as fast as those with reward but had slower behavioral responses (c.f., Georgopoulos et al., 2022). Stress generated by the associations with negative value might have caused their slower behavioral responses to neutral faces with punishment, which might also explain the absence of a relationship between positive (rewarded) neutral faces and AT severity. Future studies using eye-tracking might help to clarify this issue.

The absence of a relationship between positive neutral faces and AT severity is inconsistent with a previous visual search study demonstrating impaired detection of happy facial expressions (Sato et al., 2017). It is plausible that neutral faces with positive emotional value might not have been less emotionally evocative than the truly positive (i.e., smiling) faces among our participants with higher ATs. As such, they may have processed the neutral faces with positive emotional value similarly to the participants with low ATs. Further studies with larger samples will be necessary to clarify the modulatory effect of ATs on the detection speed for neutral faces with positive emotional value.

Some limitations to our study should be noted. First, we used only face stimuli, which might allow the possibility that if neutral objects were associated with emotional value via learning, the results would be similar to the results found in our study. However, a prior study examining attentional capture by learned neutral objects did not show a relationship between autistic traits and neutral objects associated with negative emotional value (punishment) (Anderson and Kim, 2018), suggesting that the obtained effect might be face-specific, which is consistent with the view that autistic traits involve deficits to social stimuli like faces (American Psychiatric Association, 2013). Still, future research should compare the effect of learned faces with that of learned objects on visual detection performance. Second, the link between processing innate emotional facial expressions and learned faces with emotional value remains to be clarified. Although innate emotional facial expressions are known to influence/alter the perceiver’s emotion along the dimensions of valence and arousal (Russell et al., 2003), which might be similar to the emotion experienced by the participants after successful learning of the associations of neutral faces with emotional value in our study, the underlying mechanisms by which the rapid detection of innate facial expressions and learned faces is achieved requires further clarification.

In conclusion, this study demonstrated an inverse relationship between AT severity and the detection speed for neutral faces with learned negative emotional value. This suggests that individuals with high ATs may be slow in reacting to and coping with negative faces in their daily lives, which in turn may inhibit their social–emotional interactions.

## Data availability statement

The original contributions presented in the study are included in the article/Supplementary material, further inquiries can be directed to the corresponding author.

## Ethics statement

The studies involving humans were approved by the ethics committee of the Unit for Advanced Studies of the Human Mind at Kyoto University. The studies were conducted in accordance with the local legislation and institutional requirements. The participants provided their written informed consent to participate in this study.

## Author contributions

AS: Conceptualization, Investigation, Methodology, Writing – original draft, Writing – review & editing. WS: Conceptualization, Formal analysis, Funding acquisition, Investigation, Methodology, Supervision, Writing – review & editing. SY: Conceptualization, Supervision, Validation, Writing – review & editing.
