# Supplementary material for: Brief research report: autistic traits modulate the rapid detection of punishment-associated neutral faces
Source: Front Psychol. 2023 Nov 21;14:1284739. doi: 10.3389/fpsyg.2023.1284739 (PMC10702596; doi:10.3389/fpsyg.2023.1284739)
Supplement: Supplementary file 1 [file Table_1.DOCX]

Supplementary Material

# Supplementary Data

Visual Search performance (Mean RTs, 1000=1sec) , selection rates (learning) and AQ scores (one participant’s RT difference scores for the punishment condition were excluded because they were outliers (> 3 SD from the group mean).

| No. | Gender(0=female) | Age | Education | zero-reward(ms) | zero-punishment (ms) | Reward selection rates | Punishment selection rates | AQ scores |
| --- | --- | --- | --- | --- | --- | --- | --- | --- |
| 1 | 0 | 21 | 15 | 106.90 | 94.55 | 0.8 | 0.2 | 8 |
| 2 | 0 | 23 | 17 | 73.63 | 44.02 | 0.8 | 0.3 | 11 |
| 3 | 1 | 22 | 15 | 10.74 | 82.81 | 0.8 | 0.2 | 5 |
| 4 | 1 | 23 | 14 | -53.00 | 113.19 | 0.7 | 0.2 | 7 |
| 5 | 0 | 23 | 17 | -84.11 | 20.73 | 0.8 | 0.2 | 10 |
| 6 | 1 | 23 | 17 | 134.47 | 73.39 | 0.8 | 0.2 | 7 |
| 7 | 0 | 22 | 16 | 66.40 | 76.42 | 0.8 | 0.2 | 8 |
| 8 | 0 | 20 | 14 | 69.58 | 0.30 | 0.8 | 0.2 | 9 |
| 9 | 1 | 25 | 18 | -36.22 | -29.69 | 0.8 | 0.3 | 9 |
| 10 | 1 | 21 | 15 | 129.32 | 167.66 | 0.8 | 0.2 | 9 |
| 11 | 1 | 20 | 14 | -71.29 | 16.91 | 0.8 | 0.2 | 12 |
| 12 | 0 | 21 | 15 | 85.75 | -26.75 | 0.8 | 0.2 | 11 |
| 13 | 1 | 27 | 19 | -98.19 | 145.77 | 0.8 | 0.2 | 13 |
| 14 | 1 | 23 | 17 | 265.06 | 330.77 | 0.8 | 0.2 | 8 |
| 15 | 1 | 22 | 16 | 10.61 | 45.66 | 0.8 | 0.3 | 16 |
| 16 | 1 | 22 | 16 | 77.70 | 54.02 | 0.8 | 0.3 | 8 |
| 17 | 0 | 25 | 18 | 39.39 | -71.88 | 0.8 | 0.2 | 10 |
| 18 | 1 | 22 | 16 | 44.84 | -21.77 | 0.8 | 0.2 | 11 |
| 19 | 1 | 22 | 15 | -11.02 | -6.75 | 0.8 | 0.3 | 10 |
| 20 | 1 | 20 | 14 | 94.99 | 178.54 | 0.8 | 0.2 | 8 |
| 21 | 1 | 21 | 15 | 134.32 | 54.25 | 0.8 | 0.2 | 14 |
| 22 | 1 | 21 | 14 | -68.69 | -37.37 | 0.8 | 0.2 | 6 |
| 23 | 0 | 20 | 13 | 6.90 | -42.80 | 0.8 | 0.3 | 13 |
| 24 | 0 | 19 | 13 | 97.22 | -6.02 | 0.9 | 0.5 | 8 |
| 25 | 0 | 20 | 14 | 243.58 | 90.44 | 0.8 | 0.3 | 14 |
| 26 | 0 | 22 | 16 | 8.72 | 55.77 | 0.8 | 0.3 | 12 |
| 27 | 0 | 20 | 13 | 50.97 | 61.57 | 0.9 | 0.2 | 11 |
| 28 | 0 | 23 | 17 | 217.02 | 102.30 | 0.8 | 0.2 | 16 |
| 29 | 0 | 20 | 14 | 85.51 | 99.51 | 0.8 | 0.1 | 10 |
| 30 | 0 | 23 | 15 | -33.24 | 32.55 | 0.8 | 0.2 | 12 |
| 31 | 0 | 23 | 16 | 75.36 | 136.41 | 0.5 | 0.2 | 12 |
| 32 | 0 | 21 | 15 | -30.89 | -2.42 | 0.8 | 0.3 | 15 |
| 33 | 1 | 21 | 14 | 69.42 | -22.76 | 0.8 | 0.3 | 12 |
| 34 | 1 | 21 | 15 | 50.27 | -144.54 | 0.8 | 0.2 | 5 |
| 35 | 0 | 21 | 13 | 316.55 | 224.27 | 0.8 | 0.2 | 8 |
| 36 | 0 | 22 | 15 | 74.70 | -29.69 | 0.8 | 0.2 | 10 |
| 37 | 1 | 22 | 16 | 32.80 | -0.54 | 0.8 | 0.2 | 15 |
| 38 | 1 | 20 | 15 | -57.02 | 15.02 | 0.8 | 0.3 | 9 |
| 39 | 0 | 22 | 15 | -4.73 | -77.72 | 0.8 | 0.2 | 13 |
| 40 | 1 | 20 | 13 | 66.33 | -2.50 | 0.8 | 0.2 | 10 |
| 41 | 1 | 20 | 13 | 44.96 | 85.18 | 0.8 | 0.2 | 6 |
| 42 | 1 | 21 | 16 | 69.12 | 2.25 | 0.8 | 0.3 | 10 |
| 43 | 1 | 25 | 17 | 143.66 | 111.94 | 0.8 | 0.2 | 9 |
| 44 | 0 | 24 | 18 | 142.91 | 35.45 | 0.8 | 0.2 | 15 |
| 45 | 1 | 22 | 15 | -119.49 | 22.71 | 0.8 | 0.2 | 5 |
| 46 | 1 | 21 | 15 | 44.25 | 11.03 | 0.8 | 0.2 | 9 |
| 47 | 0 | 24 | 18 | 13.33 | 91.63 | 0.8 | 0.2 | 12 |
| 48 | 0 | 22 | 16 | 47.36 | -74.25 | 0.8 | 0.2 | 17 |
| 49 | 1 | 21 | 14 | -157.70 | excluded | 0.8 | 0.2 | 11 |
| 50 | 0 | 27 | 17 | 71.70 | 156.20 | 0.7 | 0.11 | 16 |
| 51 | 1 | 23 | 16 | -2.75 | 6.51 | 0.8 | 0.2 | 10 |
| 52 | 0 | 22 | 15 | 140.35 | 46.13 | 0.8 | 0.1 | 12 |
| 53 | 1 | 24 | 17 | 141.55 | 199.43 | 0.8 | 0.2 | 8 |
| 54 | 0 | 23 | 17 | 57.33 | 24.83 | 0.8 | 0.2 | 12 |
| 55 | 1 | 25 | 19 | 0.71 | -114.61 | 0.8 | 0.2 | 16 |
| 56 | 1 | 23 | 17 | -11.33 | -42.05 | 0.8 | 0.3 | 16 |
